# Safety and Results of Bioelectrical Impedance Analysis in Patients with Cardiac Implantable Electronic Devices

**DOI:** 10.21470/1678-9741-2019-0098

**Published:** 2020

**Authors:** Luíza Matos Garlini, Fernanda Donner Alves, Adriano Kochi, Priccila Zuchinali, Leandro Zimerman, Mauricio Pimentel, Ingrid Schweigert Perry, Gabriela Corrêa Souza, Nadine Clausell

**Affiliations:** 1 Universidade Federal do Rio Grande do Sul - UFRGS, Porto Alegre, RS, Brazil.; 2 Centro Universitário Ritter dos Reis - Uniritter, Porto Alegre, RS, Brazil.; 3 Hospital de Clínicas de Porto Alegre - HCPA, Porto Alegre, RS, Brazil.; 4 Hospital Mãe de Deus, Porto Alegre, RS, Brazil.

**Keywords:** Electric Impedance. Body Water. Electrophysiologic Techniques, Cardiac. Body Composition. Electricity. Electrodes. Telemetry

## Abstract

**Objective:**

To analyze the dual interference between cardiac implantable electronic devices (CIEDs) and bioelectrical impedance analysis (BIA).

**Methods:**

Forty-three individuals admitted for CIEDs implantation were submitted to a tetrapolar BIA with an alternating current at 800 microA and 50 kHz frequency before and after the devices’ implantation. During BIA assessment, continuous telemetry was maintained between the device programmer and the CIEDs in order to look for evidence of possible electric interference in the intracavitary signal of the device.

**Results:**

BIA in patients with CIEDs was safe and not associated with any device malfunction or electrical interference in the intracardiac electrogram of any electrode. After the implantation of the devices, there were significant reductions in BIA measurements of resistance, reactance, and measurements adjusted for height resistance and reactance, reflecting an increase (+ 1 kg; *P*<0.05) in results of total body water and extracellular water in liter and, consequently, increases in fat-free mass (FFM) and extracellular mass in kg. Because of changes in the hydration status and FFM values, without changes in weight, fat mass was significantly lower (-1.2 kg; *P*<0.05).

**Conclusion:**

BIA assessment in patients with CIEDs was safe and not associated with any device malfunction. The differences in BIA parameters might have occurred because of modifications on the patients’ body composition, associated to their hydration status, and not to the CIEDs.

**Table:** 

Abbreviations, acronyms & symbols				
BIA	= Bioelectrical impedance analysis		ICD	= Implantable cardioverter defibrillator
BCM	= Body cell mass		ICW	= Intracellular water
BMI	= Body mass index		NYHA	= New York Heart Association
CIEDs	= Cardiac implantable electronic devices		PA	= Phase angle
CRT	= Cardiac resynchronization therapy		PM	= Pacemaker
CRT-D	= Cardiac resynchronization therapy defibrillator		R	= Resistance
DBP	= Diastolic blood pressure		RVI	= Right ventricle impedance
DM	= Diabetes mellitus		RVT	= Right ventricle threshold
ECM	= Extracellular mass		SAH	= Systemic arterial hypertension
ECW	= Extracellular water		SBP	= Systolic blood pressure
FFM	= Fat-free mass		SD	= Standard deviation
FM	= Fat mass		SPSS	= Statistical Package for the Social Sciences
H	= Height		TBW	= Total body water
HF	= Heart failure		Xc	= Reactance

## INTRODUCTION

The bioelectrical impedance analysis (BIA) has been described since the 1970s as a body composition assessment method based on the opposition of the biological environment to the alternating electric current, determined by the resistance (R) and reactance (Xc) components^[1,2]^. It has been used both in healthy individuals and in some clinical situations, such as malnutrition, trauma, pre- and post-surgical period, liver disease, kidney failure, and heart disease^[3-5]^. It stands out by its applicability at bedside due to the practicality of the method, compared to other body composition techniques^[6]^.

Cardiac implantable electronic devices (CIEDs) have been used in clinical practice for about five decades. Pacemaker (PM) is used to treat clinically significant bradyarrhythmia. Implantable cardioverter defibrillator (ICD) is used in primary and secondary prevention of sudden cardiac death. In patients with heart failure (HF), ICD and cardiac resynchronization therapy (CRT) can be used, individually or combined (CRT defibrillator [CRT-D)]^[7-10]^.

The body composition analysis through BIA assessment is not recommended for CIEDs carriers because it can cause an interference on the devices due to the applied electric current^[11]^. However, the small magnitude of the current sent to the body is way inferior to the susceptibility limits of the devices, such as PM and ICD. Still, there is an absence of information on the devices’ manuals about the use of BIA in CIEDs carriers.

In addition, the protocols for BIA assessment demand attention to the presence of metallic objects in order to avoid conduction of the electric current and erroneous results^[11]^, which could be relevant to CIEDs carriers. Considering the limited amount of thorough detailed safety analyses, it is recommended to avoid applying body impedance methods in these cases^[12]^. This limitation may influence not only the benefits that BIA could provide for these patients, such as prognosis and body composition, but also the exclusion of this potentially critical population from analyses of body composition and mortality. This study investigated the dual interference between CIEDs and BIA.

## METHODS

### Study Population

This is a prospective study that included adult patients (age ≥ 18 years old) from the Brazilian Public Health System referred for CIEDs (PM, ICD, and CRT-D) implantation at the Hospital de Clínicas de Porto Alegre, from June 2015 to November 2016. Exclusion criteria were presence of hydroelectrolytic disorders, edema, amputated limbs, extreme body mass index (BMI) (<16 or >36 kg/m²), skin lesions, and women in menstrual period.

The study was approved by the Ethics Committee of the Hospital de Clínicas de Porto Alegre and it was conducted according to ethical principles of Helsinki. A written informed consent was obtained from all participants before taking part in the study.

### Study Protocol

#### Anthropometric Evaluation

The weight values were obtained by a digital scale and the height (H) by a vertical stadiometer attached to a Filizola® scale - Personal Line (São Paulo, Brazil). Then, BMI was calculated by dividing weight (kg) by square H (m) (WHO, 2000)^[13]^.

#### BIA Assessment

BIA was performed with the tetrapolar device Biodynamics 450 (Biodynamics Corp., Seattle, Washington, USA), which has a 50 kHz frequency and 800 microA current, with duration of 10 to 15 seconds. The subjects were instructed to fast for at least four hours, perform no physical activity the day prior to the assessment, and remove all metallic objects in their possession^[1,14]^.

The assessments were performed with the patient in supine position, with arms apart from the body and separated legs. Four disposable electrocardiographic electrodes were used in each assessment (Conmed Corporation, Utica, NY). Two distal electrodes were positioned on the dorsal surfaces (in the hand, next to the metacarpus and the phalange joint, and the foot, next to the metatarsus and the phalange joint), and two were placed on the pisiform prominence of the wrist and between the medial lateral malleolus of the ankle^[15]^. The collected measurements of BIA were phase angle (PA), fat-free mass (FFM), fat mass (FM), body cell mass (BCM), extracellular mass (ECM), total body water (TBW), intracellular water (ICW), and extracellular water (ECW). Direct measurements of the BIA R and Xc, both also adjusted for H (R/H and Xc/H), were evaluated. BIA was performed in two moments, before and after the implantation of the CIEDs, obtaining an average of eight days (±9.2) between the first and second evaluation.

#### Evaluation of CIEDs Functioning

The CIEDs assessments were performed by a cardiac electrophysiologist with a specific device programmer designed for interrogation of the device’s parameters. The device programmer head was placed over the patient’s CIED to establish communication. Baseline interrogation was performed, including assessment of electrical impedance, sensing and capturing thresholds of all leads. In patients with ICD, while tachyarrhythmia detection and sensing were programmed on, all therapies (antitachycardia pacing and shock) were temporarily programmed off to avoid the possibility of inappropriate therapies. During the BIA assessment, continuous telemetry was maintained between the device programmer and CIED in order to look for evidence of possible electric interference in the intracavitary signal of the device. After completion of BIA, sensing and pacing parameters were measured again and all tachyarrhythmias therapies were programmed back to their original settings. The possible detection of anomalous electric activity secondary to the passage of the electric current not intrinsic to the heart on the ventricular electrogram was verified through continuous observation of the intracavitary signal on the device setting during the passage of the BIA’s electrical current in the presence of a high programmable sensitivity for that device.

### Statistical Analysis

Data are presented in mean and standard deviation (SD) or median and interquartile range. Continuous variables were compared using Student’s *t*-test for paired samples or the paired Wilcoxon test, in order to obtain the differences of the BIA parameters before and after the implantation of CIEDs, and the values of the CIEDs before and after BIA. According to the BIA’s manufacturer, a 2 to 3% error is expected due to the standardization protocol for conducting the exam.

The normality analysis was assessed by the Shapiro-Wilk test. Categorical variables were described as absolute and relative frequency. The significance level used was 5%. In order to analyze the data, the Statistical Package for the Social Sciences (SPSS) software (SPSS, Chicago) version 23.0 was used.

## RESULTS

The sample consisted of 43 individuals, most were male and Caucasian, mean age was 66 ± 10 years, and 39% of them presented HF (Table 1).

**Table 1:** Characteristics of the study population (n=43).

Characteristics	n (%); mean±SD
Gender (male)	26 (59%)
Ethnicity (Caucasian)	41 (98%)
Age (years)	66±10
SBP (mmHg)	127.5±31
DBP (mmHg)	71±15
SAH	31 (74%)
DM	13 (30%)
HF (n=17)	
NYHA I - II	12 (71%)
NYHA III - IV	5 (29%)
Ischemic etiology	10 (59%)

Data described as absolute and relative frequency, mean, and standard deviation (SD). DBP=diastolic blood pressure; DM=diabetes mellitus; HF=heart failure; NYHA=New York Heart Association; SAH=systemic arterial hypertension; SBP=systolic blood pressure

Twenty-two patients had PM implanted, 15 had an ICD, and six had CRT-D. Implanted CIEDs were manufactured by Medtronic (n=27) and Biotronik (n=16) (Table 2).

**Table 2:** Characteristics of the devices implanted in the study sample.

Characteristics	n (%)
Type	
PM	22 (51%)
Unicameral	4 (18%)
Bicameral	18 (82%)
ICD	15 (35%)
Unicameral	9 (60%)
Bicameral	6 (40%)
CRT-D	6 (14%)
CIED manufacturer	
Biotronik^®^	16 (37%)
Medtronic^®^	27 (63%)
CIED implantation	
Right side	15 (35%)
Left side	28 (65%)

Biotronik^®^ (Berlin, Germany); Medtronic^®^ (Minneapolis, USA) CIED=cardiac implantable electronic device; CRT-D=cardiac resynchronization therapy defibrillator; ICD=implantable cardioverter-defibrillator; PM=pacemaker

Regarding variations in anthropometric and BIA measurements, weight, BMI, PA, BCM, and ICW showed no difference between the first assessment, without the implanted device, and the second, with the implanted device. However, there were significant reductions (before and after CIEDs implantations) in direct BIA measurements (R, R/H, Xc, Xc/H), reflecting an increase (+ 1 kg) in results of TBW (L) and ECW (L) and, consequently, increases in FFM (kg) and ECM (kg). Because of changes in the hydration status and FFM values, without changes in weight, FM was significantly lower (Table 3). All of these variants were approximately 1.36% of the patients' weight, being within the possible margin of error, referring to the protocol to make the examination, especially if they were changes in hydration status.

**Table 3:** Comparison of the anthropometric variables and the derived variables from BIA before and after CIEDs implantation.

	Values before CIEDs implantation	Values after CIEDs implantation	*P*-value
Weight (kg)	73.4±11.0	73.3±10.8	0.941
BMI (kg/m^2^)	27.5±4.1	27.5±4.1	0.947
PA (º)	6.3 (5.6 - 7.0)	5.9 (5.5 - 6.9)	0.067
FFM (kg)	50.6±8.3	51.6±8.9	0.035
FM (kg)	22.8±8.3	21.6±8.1	0.017
TBW (L)	36.7±5.8	37.7±6.3	0.018
ICW (L)	19.7±4.1	19.7±4.3	0.940
ECW (L)	17.0±2.8	18.1±3.1	<0.001
BCM (kg)	23.3±4.9	23.2±4.9	0.764
ECM (kg)	27.3±4.6	28.4±4.7	0.003
R (Ohms)	483.8 (455.2 - 567.4)	464.3 (421.5 - 537.1)	<0.001
R/H (Ohms/m)	317.7±64.7	299.9±59.2	0.017
Xc (Ohms)	55.3±11.9	50.7±12.2	0.001
Xc/H (Ohms/m)	33.7±7.3	31±7.3	0.001

Data described as mean and standard deviation or median and interquartile interval (P25-P75). BCM=body cell mass; BIA=bioelectrical impedance analysis; BMI=body mass index; CIEDs = cardiac implantable electronic devices; ECM=extracellular mass; ECW=extracellular water; FFM=fat-free mass; FM=fat mass; H=height; ICW=intracellular water; PA=phase angle; R=resistance; TBW=total body water; Xc=reactance

The impact of BIA assessment on CIEDs showed no electrical interference on intracardiac electrogram of any lead (atrial, right ventricular, left ventricular), in any patient. There was no inappropriate sensing or tachycardia detection in any device. Full interrogation of CIEDs after BIA showed no significant differences in lead parameters or device function (Table 4). No patient presented symptoms during BIA.

**Table 4:** Comparison between the CIEDs parameters before and after the examination using BIA.

	Values before BIA assessment	Values after BIA assessment	*P*-value
RVI (Ohm)	547.5±97.1	550.3±107.8	0.679
R wave (mV)	11.6±7.0	12.0±6.8	0.225
RVT (V)	0.7±0.5	0.8±0.5	0.276

Data described as mean and standard deviation. RVI=right ventricle impedance; RVT=right ventricle threshold

## DISCUSSION

In our study, BIA with Biodynamics 450 (Biodynamics Corp. Seattle, Washington, USA) in patients with CIEDs was safe and not associated with any device malfunction. We did not find presence of electrical interference on intracardiac electrogram of any lead (atrial, right ventricular, left ventricular) in any patient. With respect to BIA measurements, the significant variations were related to body water and were within the margin of error of the examination.

Since this is a fast, safe, and non-invasive method, with a relatively low cost, the BIA assessment has been used to estimate the body composition and the nutritional status of healthy individuals and patients^[1,16]^. Nowadays, its prognostic use has been studied through PA, and a possible correlation between low PA values and mortality has been observed in patients with heart disease^[4,17]^.

Concerning the interference of BIA on CIEDs, the same findings were observed by Buch et al.^[18]^, while performing BIA at the frequencies of 5, 50, and 500 kHz in 20 individuals with HF. No evidence of interference with ICD function was seen in any patient, including no telemetry disruption, no oversensing on any lead, and no patient symptoms. In another larger study (n=63), which also found no electromagnetic interferences in ICDs, the authors conclude on the safety of this procedure and recommend that the current guidelines should be updated^[19]^. Recently, a study of the findings corroborated the results, in an analysis of 200 individuals, indicating that the use of BIA is safe in patients with cardiac devices^[20]^.

Despite no interferences being observed on the devices in these previous studies^[18,19]^, they did not assess the BIA parameters, which can also suffer interferences, since they are wrapped by a metallic housing. The instructions regarding the use of BIA include the removal of metallic objects, attention so that the patient is not in contact with metallic structures and, if there is presence of any sort of prosthesis, BIA examination must be on the opposite side in order to avoid conduction of the electric current^[11]^.

In BIA measurements, an increment in TBW was observed, which may have occurred by an increase in the hydric volume due to the conditions in the hospital stay, such as saline solution administration after the procedure, and other external factors. This increase in TBW value seems to have been due to the increase in ECW (*P*<0.001) and for this reason there was no change in FFM (which contains large amounts of ICW ~73%). Another measure of BIA affected by hydration was the ECM, that reflects all the metabolically inactive (non-living) parts of the body, including water contained outside living cells. Still, since resistance values (R and R/H) correlate highly with fluid balance, by facilitating the passage of the electric current, the increase of TBW can impact in the reduction of R and R/H^[21]^. Similar results were found by Pinto et al.^[22]^ in 62 patients with an indication for PM or defibrillator implantation, and they concluded that decrease in resistance and related parameters were associated to hydration status^[2]^. This limitation may influence not only the benefits that BIA could provide for these patients, such as prognosis and body composition, but also the exclusion of this potentially critical population from analyses of body composition and mortality. This study analyzed the dual interference between CIEDs and BIA^[22]^.

A reduction in reactance values (Xc and Xc/H), reflecting tissue (not water) changes or the absorption of electrical current in tissues, was also demonstrated in our study, and this reflected in the reduction of the calculated measurement in BIA of FM.

Our study has some possible limitations that need to be considered. Our sample was relatively small, and we studied only a single BIA system in patients with CIEDs of two manufacturers. Considering these, our results cannot be applied to all patients with CIEDs or to different BIA systems. Another limitation is the variability of the evaluation time between the first and second BIA. This interval might have influenced the body composition of the individuals, as it was observed in the FM and TBW outcomes, in which case CIEDs might not have influenced this finding.

## CONCLUSION

In our study, BIA assessment in patients with CIEDs was safe and not associated with any device malfunction. The differences in BIA measurements might have occurred because of modifications on the patients’ body composition, caused by their hydration status, and not by the CIEDs’ presence. Further larger studies are required to confirm these findings and to determine a change in guidelines and manufacturer’s recommendations.

**Table:** 

Author's roles & responsibilities	
LMG	Substantial contributions to the conception of the work, analysis, and interpretation of data for the work; drafting the work and revising it critically for important intellectual content; final approval of the version to be published
FDA	Substantial contributions to the conception of the work; final approval of the version to be published
AK	Substantial contributions to the conception of the work, analysis, and interpretation of data for the work; drafting the work and revising it critically for important intellectual content; final approval of the version to be published
PZ	Drafting the work or revising it critically for important intellectual content; final approval of the version to be published
LZ	Drafting the work or revising it critically for important intellectual content; final approval of the version to be published
MP	Substantial contributions to the interpretation of data for the work; drafting the work and revising it critically for important intellectual content; final approval of the version to be published
ISP	Substantial contributions to the interpretation of data for the work; drafting the work and revising it critically for important intellectual content; final approval of the version to be published
GCS	Substantial contributions to the interpretation of data for the work; drafting the work and revising it critically for important intellectual content; agreement to be accountable for all aspects of the work in ensuring that questions related to the accuracy or integrity of any part of the work are appropriately investigated and resolved; final approval of the version to be published
NC	Drafting the work or revising it critically for important intellectual content; agreement to be accountable for all aspects of the work in ensuring that questions related to the accuracy or integrity of any part of the work are appropriately investigated and resolved; final approval of the version to be published
